# Whose education matters for later-life health trajectories? A three-generation comparison in China

**DOI:** 10.3389/ijph.2026.1609298

**Published:** 2026-05-20

**Authors:** Tingshuai Ge, Qing Han

**Affiliations:** School of Public Policy and Administration, Xi’an Jiaotong University, Xi’an, China

**Keywords:** China, education, gender differences, health inequality, later life

## Abstract

**Objectives:**

This study examined the effects of own, parental, spousal, and children’s education on later-life health trajectories in the Chinese context, exploring variations across health measures and genders in these processes.

**Methods:**

Hierarchical linear regression models were employed to the data from China Health and Retirement Longitudinal Study over the period 2011–2020 (N = 15,304 individuals aged 45–85; N = 62,836 person-years).

**Results:**

Among men, mental health disparities by own education remained stable with age, while those linked to spousal and children’s education widened. For physical health, disparities by own education were stable, whereas those related to children’s education diminished with age. Among women, mental health disparities tied to own and family members’ education widened with age. Physical health disparities driven by own and children’s education increased with age, while those associated with spousal education remained stable.

**Conclusion:**

Our findings suggest that family members’ education differentially shapes later-life health trajectories, with these processes being sensitive to health measures and gender. Policies that enhance women’s education and support disadvantaged families and are essential to reduce health inequalities among aging populations.

## Introduction

Numerous studies have demonstrated the health benefits of education across diverse contexts, including the United States [1, 2], Europe [3], and China [4], through socioeconomic and behavioral pathways [2, 5]. Some evidence further suggests that these benefits may strengthen with age [6, 7]. However, significant gaps persist in understanding the association between education and health.

First, the extent to which health benefits accrue from other family members’ education, beyond an individual’s own, remains underexplored. Later-life health is shaped not only by individuals’ own education but also by resources embedded in family relationships across the life course. For example, parental education lays an important foundation for health over the life course: well-educated parents can provide nutritious meals and foster healthy habits during childhood [8, 9]; individuals with higher-educated parents are also more likely to attain higher levels of education and secure better occupations in adulthood [10, 11], and may face fewer caregiving burdens in mid-to-later life because their parents tend to be in better health [12]. After marriage, individuals may also benefit from the social, economic, psychological, and behavioral resources associated with having a highly educated spouse, which can help mitigate or prevent adverse health consequences [13, 14]. In later life, children’s education may likewise play an important protective role by helping parents alleviate financial strain, access high-quality healthcare, and adopt healthier behaviors [15, 16]. Although existing studies have begun to examine the spillover effects of parental [12, 17], spousal [5, 13], and children’s education [10, 15, 18] on individual health, these family members are often considered separately, or the focus remains primarily on individuals’ own education. As a result, the full extent of social stratification in health may be underestimated [14].

Second, it remains unclear whether the health benefits associated with family members’ education increase, persist, or diminish with age. Two opposing theoretical perspectives, the cumulative advantage/disadvantage (CAD) hypothesis [19] and the age-as-leveler (AL) hypothesis [20], offer different expectations regarding how education-related health disparities evolve over the life course. Applied to the education of family members, these perspectives suggest that health disparities may widen with age if educational resources provided by family members accumulate over time, or narrow with age if biological decline or institutional protection reduces the importance of such resources in later life. Nevertheless, prior studies have focused largely on average health outcomes rather than health trajectories [5, 10, 17]. Compared with examining average health levels alone, analyzing health trajectories can more directly reveal how family educational resources shape health disparities across the life course, while also extending the applicability of the CAD and AL hypotheses to the dynamic development of health inequality.

Third, the influence of family members’ education on health trajectories may vary by health measure and gender. For health measures, different dimensions of health may differ in their sensitivity to the social resources provided by family members. Physical health may depend more on material resources such as financial support, whereas mental health may be more responsive to non-material resources such as emotional comfort [21]. In addition, different dimensions of health may follow distinct age-related patterns of decline [22, 23]. Gender may also modify the association between family members’ education and health trajectories. Gender role norms in many societies position men as breadwinners and require women to be the caregivers and kin-keepers [10]. On the one hand, this may place women at a disadvantage in terms of access to material resources and expose them to greater work–family conflict [24, 25]. On the other hand, it may lead women to maintain closer ties with other family members than men do [10]. As a result, women’s health may be more strongly shaped by the educational resources of both themselves and other family members. However, prior findings on health inequality by education remain highly inconsistent [22, 23]. One way to address this limitation is to complement physical health indicators with measures of mental health and to explicitly consider gender differences.

Lastly, strong intergenerational obligations and reciprocal dependence among family members, coupled with significant educational expansion across generations, may produce distinct patterns of health disparities in China as compared to Western contexts. Most existing evidence on multigenerational education and later-life health comes from Western settings, especially the United States and Europe. These contexts are generally characterized by greater emphasis on individual independence and by more developed welfare and formal care systems. However, China differs in several respects. First, Chinese family culture emphasizes intergenerational responsibility and interdependence [26, 27]. This may imply that education-related health resources are transmitted more closely within Chinese families, making individuals’ health trajectories more likely to be shaped by the education of other family members. Second, the traditional norm of raising children for old-age support (“*Yang Er Fang Lao*”) has long been embedded in Chinese family culture. Despite profound social change, this tradition may persist because the social security system remains insufficiently developed in some respects. This suggests that children’s education may play a particularly important role in shaping health trajectories in later life. Third, China has experienced dramatic educational expansion across cohorts, meaning that the value and social meaning of education may differ substantially across generations. As a result, the influence of multigenerational education on health trajectories may be confounded by substantial cohort effects. Taken together, these features suggest that theoretical expectations derived mainly from Western contexts may not apply uniformly to China, and that studying the Chinese case can provide new insight into whether, to what extent, and whose education matters for health trajectories in later life.

Only a few studies to date have examined the impacts of multiple family members’ education on health trajectories in later life [9, 24, 28]. For instance, using data on older adults in Mexico, Gutierrez et al. found that higher levels of education among respondents, their parents, and their children were associated with slower declines in cognitive function [9]. A study in the United States reached a similar conclusion regarding one’s own and children’s education [28]. However, both studies focused exclusively on changes in cognitive function over survey time, overlooking the role of spousal education as well as variations by age, gender, and health dimensions. To our best knowledge, only one study has examined the effects of one’s own education and that of their parents, spouses, and children on health trajectories [24]. Still, this study focused only on depressive symptoms trajectories by children’s education, and the data used in that study, spanning 1996 to 2011, are now relatively dated and may not capture more recent patterns. Most importantly, none of these three studies adequately addressed cohort effects, despite substantial changes in educational attainment and its social meaning across birth cohorts. Prior research has identified cohort effects as an important source of inconsistency in the relationship between age and health [29].

Addressing these gaps, this study investigates how own, parental, spousal, and children’s education shape later-life mental and physical health trajectories and gender differences within these processes in the Chinese context. In doing so, the study extends the literature that has focused predominantly on Western societies to a rapidly changing developing-country setting, provides a more comprehensive family-based account of educational inequality in health, and offers a life-course perspective on whether such disparities widen, persist, or narrow with age. Specifically, using longitudinal data from the China Health and Retirement Longitudinal Study (CHARLS), following 15,304 individuals across 62,836 panel observations between 2011 and 2020, we aim to: (1) assess the influence of own, parental, spousal, and children’s education on later-life health trajectories; (2) investigate how these processes vary across mental and physical health measures; (3) evaluate whether men and women benefit equally from family members’ education.

## Methods

### Data

Data used in this study was from the CHARLS, a nationally representative survey of Chinese adults aged 45 years and above. The baseline survey was conducted in 2011 and surveyed 17,705 participants, with follow-up surveys in 2013, 2015, 2018, and 2020 [30]. All information were collected via face-to-face interviews, with a follow-up rate exceeding 80%.

We used the CHARLS data from 2011 to 2020. Our sample was comprised of participants aged 45 to 85 who were ever married and had at least one child aged 25 or older. To minimize selection bias due to mortality and morbidity, we first excluded individuals older than 85. We also excluded those never married or with children under 25 to address potential reverse causality, as participants’ poor health may influence their children’s educational attainment [9, 31]. Finally, we excluded participants with missing data on variables of interest, including Lawton Instrumental Activities of Daily Living Scale (IADL), gender, and own education. The proportion of missing values for these variables was approximately 1%. However, participants with missing 10-item Center for Epidemiologic Studies Depression Scale (CESD) or other family members’ education were not excluded solely on this basis. Given the relatively high proportions of missing values for CESD scale and other family members’ education, 9.03% and 12.53%, respectively, missing values for these variables were imputed in the main analysis. The final analytic sample included 15,304 participants with 62,836 observations. Sample restrictions are detailed in Supplementary Table SA1.

### Measures

In this study, health was assessed along two dimensions, mental health and physical health. These dimensions were approximated by depressive symptoms and instrumental activities, respectively. We used the CESD scale for measuring depressive symptoms and the IADL scale for instrumental activities. CESD and IADL were selected because they capture two distinct yet important dimensions of health in later life. CESD is not only a key indicator of mental health, but also serves as a screening test for depression in the general population, and has been validated and used in older adults [32]. IADL reflects individuals’ ability to live independently and perform instrumental daily activities and is widely used as an indicator of physical functioning among older adults. Both are widely recognized as important determinants of quality of life and survival in later life. In addition, depression and disability have become major public health concerns in the context of rapid population aging in China. It has been projected that the number of disabled older adults in China will increase from 24.85 million in 2020 to 54.72 million in 2050 [33]. Meta-analytic evidence further suggests that the prevalence of depressed older adults in China exceeds 20%, higher than that reported in many developed countries [34].

*Depressive symptoms* were assessed using the CESD scale in CHARLS. Participants were asked about the frequency of many feelings and behaviors during the last week, with four responses ranging from “none of the time” (0) to “most of the time” (3) for each item. The depressive symptoms score (0–30) was obtained by calculating the total score of ten items, with a higher score indicating worse mental health (Cronbach’s alpha ≥0.76 across 2011–2020 waves).

*Instrumental activities* were assessed considering the following activities measured with the IADL scale: doing household chores, preparing hot meals, shopping for groceries, managing assets, and taking medications. Responses ranged from “do not have any difficulty” (1) to “cannot do it” (4), producing a total score (5–20), with higher scores indicating worse physical health (Cronbach’s alpha ≥0.83 across 2011–2020 waves). Given that depressive symptoms and instrumental activities are frequently referred to in the following sections, we use CESD and IADL as shorthand terms for them throughout the remainder of the manuscript. Moreover, because these health indicators were measured on different scales, we standardized the scores of CESD and IADL to allow for a direct comparison between these indicators. It should be noted that this standardization does not imply that IADL and CESD are conceptually equivalent; rather, it places them on a common statistical scale, allowing estimates to be interpreted in standard deviation units, a method widely used in previous studies [23].

*Age and birth cohort* Age was assessed as a time-varying variable ranging from 45 to 85, and was minimum-centered. Birth cohort was included as a time-constant variable, spanning from 1926 to 1966, and was also minimum-centered.

*Family members’ education* was measured as the highest level of educational attainment achieved by family members. Parental education was based on the higher attainment of either parent, spousal education on the current or most recent spouse, and children’s education on the highest level among all children. This measurement strategy was used to capture the highest level of educational resources embedded in each intergenerational family network. It is consistent with previous studies that have used the highest educational attainment among adult children to measure children’s education in relation to older parents’ health, cognition, or dementia risk [9, 10, 35], as well as studies that have used the higher educational attainment of either parent to represent parental education or family-of-origin educational resources [9, 17, 28]. To account for the significant educational expansion in Chinese society over the past decades, which has devalued certain credentials, we categorized family members’ education into low, medium, and high levels by their birth cohorts. Specifically, family members are first grouped into ten-year birth cohorts. The older parent’s or the oldest child’s cohort was used when education levels were equal [10]. Within each ten-year cohort, the cutoffs for low, medium, and high education were determined based on two criteria: (1) major historical developments in China’s educational system, such as the 9-year compulsory education in the 1980s and the higher education expansion launched in 1999; and (2) prior studies that used similar cohort-specific classification strategies [25, 36]. The distribution of educational attainment by cohorts is presented in Supplementary Table SA2.

*Covariates* include marital status and parental alive status. Marital status was coded as 1 if the participant currently had a spouse, and 0 otherwise. Parental alive status was coded as 1 if at least one of the participant’s parents was alive, and 0 otherwise. Descriptive statistics for all variables are presented in Table 1.

**TABLE 1:** Sample characteristics (China, 2011–2020).

Variables^a^	Men (N = 29,582)		Women (N = 33,254)		Gender differences (p-value)^d^
	Mean/%	SD	Mean/%	SD	
Participant characteristics^b^					
Own education	​	​	​	​	0.000
Low	41.65%	​	69.25%	​	​
Medium	35.29%	​	18.96%	​	​
High	23.07%	​	11.79%	​	​
Parental education	​	​	​	​	0.203
Low	62.00%	​	63.39%	​	​
Medium	25.31%	​	24.21%	​	​
High	12.69%	​	12.40%	​	​
Spousal education	​	​	​	​	0.000
Low	70.43%	​	41.98%	​	​
Medium	18.66%	​	35.03%	​	​
High	10.91%	​	22.98%	​	​
Children’s education	​	​	​	​	0.266
Low	49.66%	​	48.32%	​	​
Medium	29.01%	​	29.51%	​	​
High	21.33%	​	22.17%	​	​
Marital status	​	​	​	​	0.000
Having a spouse	92.11%	​	83.57%	​	​
No spouse	7.89%	​	16.43%	​	​
Parental alive status	​	​	​	​	0.148
Alive	21.70%	​	20.74%	​	​
No parents alive	78.30%	​	79.26%	​	​
Birth year	1950.32	9.12	1951.14	9.35	0.000
Observation characteristics^c^					
Age	63.85	8.76	63.11	8.95	0.000
CESD	7.40	5.83	9.66	6.74	0.000
IADL	5.96	2.67	6.27	2.81	0.000
Number of waves	2.73	1.37	2.77	1.38	0.001

^a^ Missing values were not imputed.

^b^ Time-constant variables are summarized at the 2011 baseline.

^c^ Time-varying variables are summarized over all observations.

^d^ *Chi-square* test for categorical variables and *t*-test for continuous variables were conducted to examine the gender difference in sample characteristics. Non-standardized values of CESD, and IADL, were used; CESD, Depressive symptoms; IADL, Instrumental Activities of Daily Living; SD, standard deviation.

### Analytic strategy

We used hierarchical linear regression models (HLM) to estimate the impacts of family members’ education on health trajectories, accounting for varying intercepts and slopes across individuals. In the following analyses, we first examined the association between family members’ education and average health levels in response to previous studies. Then, we explored the impacts of family members’ education on health trajectories. By incorporating interactions between education and age, the models allowed us to assess differences in health trajectories across educational groups. In addition, to reduce the potential confounding by cohort, we followed prior research by including cohort, as well as interactions among age, cohort, and education, in the models [29]. Previous studies have shown that Chinese men and women differ substantially over the life course in educational opportunities and attainment, exposure to stressful events, access to resources, and health trajectories [25, 37]. Accordingly, all analyses were conducted separately for men and women.

In a preliminary analysis, we identified the parametrizations of age and cohort effects as well as their interactions with education on CESD and IADL. This process was guided by three methodological criteria commonly used in prior studies [23, 37]: (a) similarity between observed data (see Supplementary Figures SA1, SA2) and fitted data examined by diagnostic plots, (b) Bayesian Information Criterion (BIC), and (c) model parsimony if models were similar on criterion (a) and did not differ by more than 10 BIC points [38]. Overall, model fit analyses suggested slightly different model specifications, most commonly including linear and squared terms of age, linear term of cohort, interaction terms between linear terms of age and education, and interaction terms between linear terms of cohort and education (see Table 2 and Supplementary Tables SA7 and SA8 for details).

**TABLE 2:** Results of the hierarchical linear model for average level of health outcomes (China, 2011–2020).

Variables	CESD				IADL			
	M1: Men		M2: Women		M3: Men		M4: Women	
	β	SE	β	SE	β	SE	β	SE
Age	−0.036^*^	(0.014)	−0.018	(0.015)	−0.011^***^	(0.003)	−0.002	(0.003)
Age^2^	0.001^***^	(0.000)	0.001^*^	(0.000)	0.001^***^	(0.000)	0.001^***^	(0.000)
Cohort	−0.011	(0.006)	−0.000	(0.006)	−0.004^*^	(0.002)	−0.001	(0.002)
Age # cohort	0.001^***^	(0.000)	0.001^***^	(0.000)	​	​	​	​
Own education (ref. Low)								
Medium	−0.095^***^	(0.021)	−0.168^***^	(0.025)	−0.056^***^	(0.014)	−0.075^***^	(0.018)
High	−0.221^***^	(0.025)	−0.365^***^	(0.032)	−0.073^***^	(0.016)	−0.130^***^	(0.019)
Parental education (ref. Low)								
Medium	−0.029	(0.022)	−0.049^*^	(0.024)	−0.027	(0.015)	−0.024	(0.015)
High	−0.054	(0.028)	−0.070^*^	(0.033)	−0.017	(0.022)	−0.015	(0.022)
Spousal education (ref. Low)								
Medium	−0.048	(0.027)	−0.037	(0.025)	−0.017	(0.016)	−0.026	(0.017)
High	−0.090^**^	(0.031)	−0.097^**^	(0.031)	−0.024	(0.016)	−0.055^**^	(0.019)
Children’s education (ref. Low)								
Medium	−0.089^***^	(0.024)	−0.129^***^	(0.023)	−0.026	(0.017)	−0.077^***^	(0.015)
High	−0.174^***^	(0.025)	−0.200^***^	(0.027)	−0.039	(0.019)	−0.088^***^	(0.021)
No spouse	0.292^***^	(0.040)	0.173^***^	(0.031)	−0.002	(0.024)	−0.032	(0.021)
No parents alive	0.040	(0.022)	0.031	(0.024)	0.011	(0.013)	0.007	(0.015)
Constant	0.225	(0.227)	0.085	(0.233)	−0.082	(0.063)	−0.096	(0.070)
Observations	26,532	​	29,610	​	26,532	​	29,610	​

CESD, Depressive symptoms; IADL, Instrumental Activities of Daily Living; SE, standard errors.

^*^ *p* < 0.05.

^**^ *p* < 0.01.

^***^ *p* < 0.001.

In this study, missing cases accounted for 22.64% of the sample, due to other family members’ education (12.53%) and CESD (9.03%). Missing education data were more prevalent among men, younger individuals, and those with higher own education and lower CESD and IADL scores, while missing CESD data were more common among women, older individuals, and those with lower family education and higher IADL scores (see Supplementary Table SA3). Thus, in our main analyses, we imputed missing values for other family members’ education and CESD using multiple imputation (20 imputations) via the *mi* command in Stata. Auxiliary variables included in the imputation model were IADL, age, cohort, gender, and own education. We adopted the “multiple imputation, then deletion” (MID) method, which involves generating imputed values and subsequently deleting observations with imputed dependent variables. This approach has demonstrated its capacity to yield more precise standard error estimates and greater robustness compared to conventional multiple imputation [39], and has been widely used in previous research [18, 24, 40]. After deleting observations for which the CESD was imputed, our analytic sample included 56,142 observations, representing 14,882 participants. We further assessed the sensitivity of our results to imputation strategies by: (1) expanding the set of auxiliary variables in the imputation model. Specifically, when imputing missing CESD values, we additionally included self-rated health, number of chronic conditions, childhood health status, smoking, drinking, and hukou status; when imputing missing values for other family members’ education, we additionally included hukou status. (2) retaining observations with imputed CESD values. The results showed minimal substantive differences (available upon request).

Around 13% of CHARLS participants were lost due to panel attrition, with higher dropout rates among men, older individuals, those with higher family education, and those with higher CESD and IADL scores (see Supplementary Table SA4). Probit models showed that participants with higher IADL scores in the previous period were more likely to drop out in the next period, though effects were small (*R* ^*2*^ ≤ 0.03; see Supplementary Tables SA5, SA6). To correct for the potential selective attrition bias, we applied the Inverse Probability Weighting (IPW) in our main analyses. Variables included in the models to calculate IPWs were CESD, IADL, age, family members’ education, and their interactions measured at *t*-1.

Lastly, we conducted several additional analyses to examine the robustness of our results. These included controlling for covariates that may affect health trajectories, testing for the potential period effects, and using an alternative measure of parental and children’s education. These analyses are presented following our primary results and can be found in Supplementary Table SA13; Supplementary Figure SA3–A9. All analyses were performed using Stata Version 17.

## Results

### Education and average health levels

Table 2 presents the associations between family members’ education and average health levels. Higher own, spousal, and children’s education were associated with lower CESD scores for both men and women, while parental education negatively influenced CESD scores only for women. For IADL, higher own education was linked to lower scores for men, whereas higher own, spousal, and children’s education were negatively associated with IADL scores for women. Parental education showed no significant association with IADL scores for either gender.

### Education and health trajectories

**FIGURE 1:**
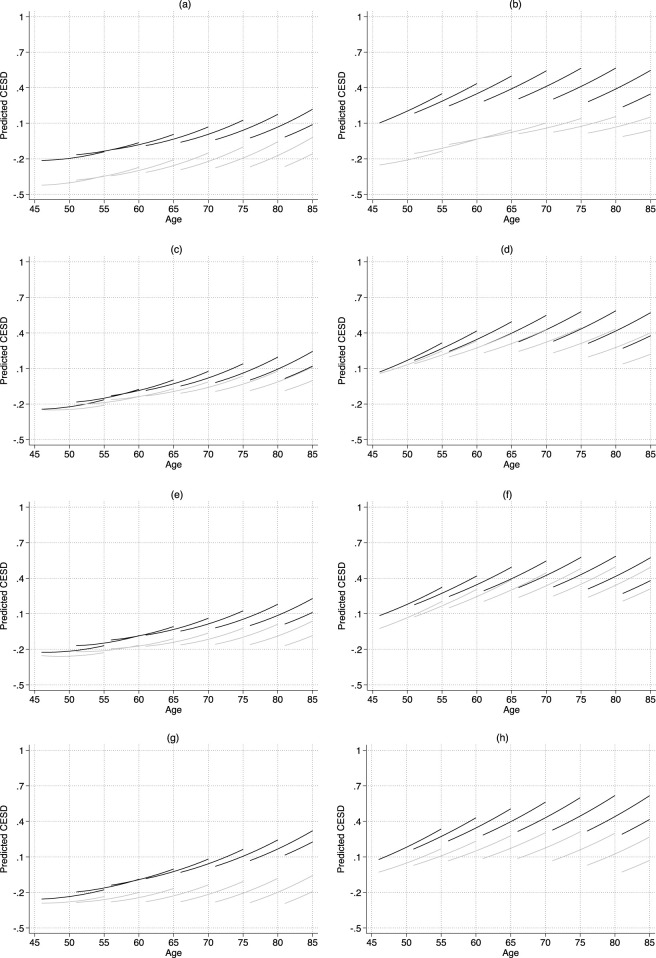
Predicted age trajectories of CESD-by-education (China, 2011–2020). Notes: CESD = Depressive symptoms. The estimates are based on Models 1–8 shown in Supplementary Table SA7. In each panel, black lines show the trajectories of people with low education, while gray lines show the trajectories of people with high education. **(a)** Own education: Men. **(b)** Own education: Women. **(c)** Parental education: Men. **(d)** Parental education: Women. **(e)** Spousal education: Men. **(f)** Spousal education: Women. **(g)** Children’s education: Men. **(h)** Children’s education: Women.

**FIGURE 2:**
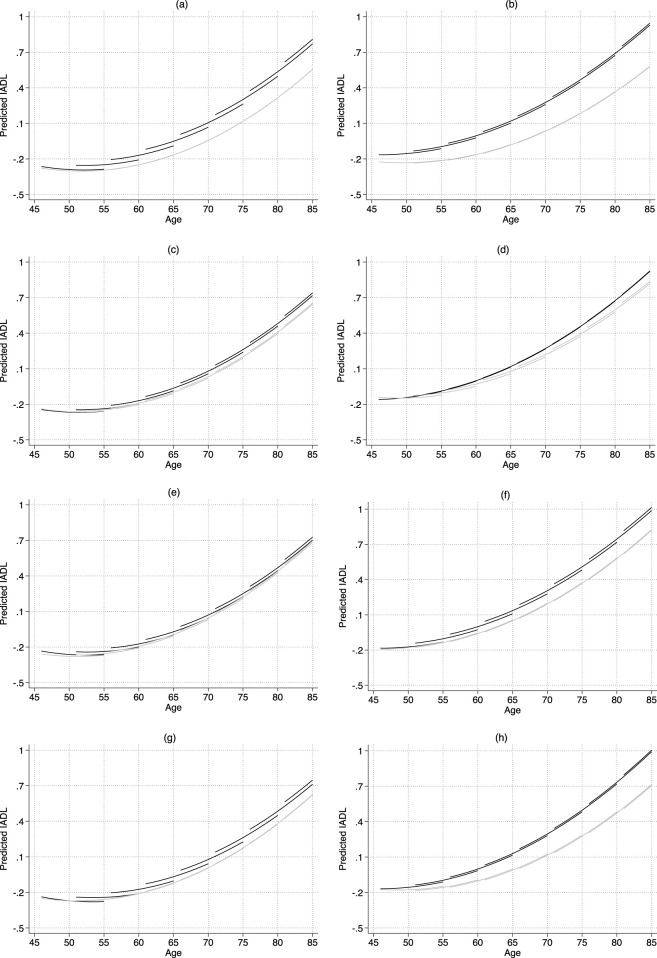
Predicted age trajectories of IADL-by-education (China, 2011–2020). Notes: IADL = Instrumental Activities of Daily Living. The estimates are based on Models 9–16 shown in Supplementary Table SA8. In each panel, black lines show the trajectories of people with low education, while gray lines show the trajectories of people with high education. **(a)** Own education: Men. **(b)** Own education: Women. **(c)** Parental education: Men. **(d)** Parental education: Women. **(e)** Spousal education: Men. **(f)** Spousal education: Women. **(g)** Children’s education: Men. **(h)** Children’s education: Women.

Supplementary Tables SA7, SA8 present our estimates of the associations between family members’ education and CESD and IADL trajectories. To facilitate interpretation, these findings are illustrated in Figures 1, 2. To better illustrate the effects of education on health trajectories after accounting for cohort effects, we present the estimated results graphically for eight birth cohorts (1930, 1935, 1940, 1945, 1950, 1955, 1960, and 1965). To evaluate age patterns and effect sizes in more detail, we calculated the corresponding marginal effects for educational differences in CESD and IADL at the age of first observation and 9 years later, controlling for cohort (see Table 3 and Supplementary Tables SA9–SA12).

**TABLE 3:** Estimated 9-year change in educational differences in Center for Epidemiologic Studies Depression Scale (CESD) and Instrumental Activities of Daily Living Scale (IADL), by gender and cohort (China, 2011–2020)^a^.

Education	Men			Women		
	Initial difference	9 years later	Change over 9 years	Initial difference	9 years later	Change over 9 years
*Panel A: CESD*						
Own education	0.226	0.220	−0.006	0.310	0.440	+0.130
Parental education	0.046	0.089	+0.043	0.064	0.119	+0.055
Spousal education	0.095	0.125	+0.030	0.089	0.100	+0.011
Children’s education	0.195	0.218	+0.023	0.198	0.258	+0.060
*Panel B: IADL*						
Own education	0.119	0.111	−0.008	0.184	0.222	+0.038
Parental education	0.033	0.037	+0.004	0.018	0.068	+0.050
Spousal education	0.031	0.012	−0.019	0.090	0.081	−0.009
Children’s education	0.064	0.037	−0.027	0.126	0.157	+0.031

^a^ Due to space constraints, this table reports summary point estimates averaged across all cohorts, including average initial differences, average differences 9 years later, and average changes over 9 years. Statistical inference should be based on the cohort-specific estimates and 95% confidence intervals reported in Supplementary Tables SA9–SA12, whereas Table 3 should be interpreted as a compact summary of the overall pattern across cohorts. Estimates are average marginal differences in standard deviations between low- and high-education groups based on Supplementary Tables SA7, SA8. Detailed cohort-specific results and their 95% confidence intervals are shown in Supplementary Tables SA9–SA12. Initial differences are predicted mean differences in CESD, or IADL, at the age of first observation. Changes over 9 years are calculated as differences between predicted mean differences at initial observation and predicted mean differences 9 years later. Differences in the gray shadow indicate that they are not statistically significant across all ages. CESD, Depressive symptoms; IADL, instrumental activities of daily living.

Three primary findings emerged from the analyses. First, CESD disparities across the later life course varied by family members’ education (see Figure 1; Table 3). For men, disparities in CESD associated with spousal and children’s education widened with age, whereas differences linked to their own education remained relatively stable over time. Specifically, for spousal education, the average CESD difference between higher and lower education levels was 0.095 at the initial observation age, increasing to 0.125 9 years later, a rise of 0.03 or 3% of a standard deviation. For children’s education, the difference increased by 2.3% of a standard deviation. In contrast, the difference associated with own education decreased slightly by 0.6% of a standard deviation, indicating stability with age. Although parental education-related CESD disparities increased with age, they remained statistically insignificant across all ages (see Supplementary Table SA10). For women, CESD disparities widened with age across all family education types. Specifically, CESD inequalities increased by 13% of a standard deviation for their own education, 6% for children’s education, 5.5% for parental education, and 1.1% for spousal education as age progressed.

Second, IADL trajectories by family members’ education differed from CESD patterns (see Figure 2; Table 3). For men, IADL disparities linked to own education remained relatively stable with age, changing by only 0.8% of a standard deviation. In contrast, disparities associated with children’s education decreased with age, with a reduction of 2.7% of a standard deviation. However, IADL differences related to parental and spousal education remained statistically insignificant throughout later life (see Supplementary Tables SA10, SA11). For women, IADL disparities associated with own and children’s education widened with age, increasing by 3.8% and 3.1% of a standard deviation, respectively. Differences linked to spousal education remained relatively stable, decreasing by a modest 0.9% of a standard deviation. Consistent with our result for men, parental education had no significant effect on women’s IADL over the later life course.

Third, the associations between family members’ education and later-life health trajectories differed between men and women. For men, CESD disparities were more strongly associated with spousal and children’s education, showing a divergence pattern with increasing age. However, higher levels of their own and children’s education were not associated with a slower deterioration in IADL, and the corresponding IADL disparities narrowed over time. For women, both CESD and IADL disparities were predominantly associated with their own and children’s education, and these disparities widened with age.

### Robustness checks

#### Controlling for covariates

We adjusted for covariates potentially influencing health trajectories, including household registration status, health behaviors, and health status. Additionally, IADL limitations was controlled in CESD models, and depression status was included in IADL models. Measures of covariates are detailed in Supplementary Table SA13. Taking health status as an example, including covariates primarily affected average health levels within education group, but our conclusions regarding patterns of health inequality remain consistent (see Supplementary Figures SA3, SA4).

#### Potential periodic confounding

Although this study focused on life-course changes in health within cohort, period effects may influence these processes. Previous research suggested that for studies with a short time span (10 years), it is reasonable to assume that there is no period effect [41]. Nonetheless, we acknowledge that periodic effects can be present and employed visual analyses to examine the potential presence of periodic trends. Our analyses revealed that CESD scores across all cohorts increased in 2015 and 2018 (see Supplementary Figure SA5). Due to the collinearity among age, cohort, and period, we included a dummy variable (1 = 2015 or 2018; 0 = other) in all models to account for potential period effects, following O’Brien [41]. Our conclusions remain unchanged (see Supplementary Figures SA6, SA7).

#### Alternative measure of parental and children’s education

Referring to previous studies [15, 42], we also utilized parental average educational attainment and the average educational attainment of all children as an alternative measure of parental and children’s education, and our results keep unchanged (see Supplementary Figures SA8, SA9).

## Discussion

A growing body of research underscores the health benefits of other family members’ education beyond an individual’s own. Yet, how these benefits evolve with age and vary by health measure and gender remain underexplored. This study advances this field by concurrently examining the influence of one’s own, parents’, spouse’s, and children’s education on later-life health trajectories, while considering heterogeneity across mental and physical health dimensions and genders in a developing country undergoing rapid social transformation. Utilizing three-generation data from CHARLS (2011–2020) and hierarchical linear models, we demonstrate that family members’ education significantly shapes age trajectories of health in later life, with considerable variations observed across health measures and genders.

We found that health benefits accrue not only from an individual’s own education but also from that of other family members, consistent with prior research on own education [4], parental education [11], spousal education [5, 43], and children’s education [18, 35]. Extending beyond these studies, we identified significant heterogeneity by health measure and gender. Notably, parental education only significantly influences women’s average CESD scores, with no effect on IADL for either gender. Similarly, higher spousal and children’s education was associated with lower IADL scores for women but showed no significant impact on men’s physical functioning.

Health disparities by own education increased with age among women but remained stable among men, aligning with previous studies in Western countries and China. For instance, based on German panel data, Leopold [23] identified a contrasting pattern in education-related differences in grip strength: disparities widened substantially with age among women, whereas they converged with age among men and showed minimal educational differences in later life. A 16-year longitudinal study in China reported comparable patterns [44]. Prior research has documented that higher education is typically associated with greater material and non-material resources [5, 42]. Women, often socially disadvantaged, are particularly dependent on educational resources to sustain health, leading to widening health gaps in later life. Conversely, men with higher education and greater resource access experience less pronounced health benefits from their own education [44].

Differences in health associated with parental education increased with age for all genders. Prior research on physical impairment, cognitive function, or general health also observed a similar trend [9, 12, 42]. In one’s early life, highly educated parents are more likely to promote them healthy behaviors and positive personality traits [8, 45]. In mid-to-late life, well-educated parents were found to continue to provide them with various forms of support [46]. Conversely, parents with lower education face a higher risk of health issues and require more care and financial support [12], which can negatively impact one’s health conditions [47]. However, we found that only women’s CESD differences by parental education were statistically significant across all ages. This suggests that early socioeconomic conditions exert a more enduring and pronounced impact on women’s mental health, aligning with prior findings [48, 49].

Mental health consistently benefits from spousal education as age progresses for all genders, while physical health does not. Prior research suggests that marriage primarily provides men with socioemotional resources and women with economic resources, both critical for maintaining mental health [13]. Consequently, CESD disparities associated with spousal education widened with age for all genders. However, we found that spousal education did not significantly affect men’s IADL disparities across ages. Compared to husbands, wives’ lower socioeconomic status may restrict their ability to provide the material resources necessary to delay the deterioration of husbands’ physical function. Additionally, as husbands’ physical function declines, the wives, often acting as primary caregivers, also experience an impact on their own health. This is evidenced by our results showing that women’s CESD disparities by spousal education become statistically non-significant around age 75, while IADL disparities by spousal education narrow slightly with advancing age (see Supplementary Table SA11).

Children’s educational differences in all genders’ mental health and women’s physical health increased with age, while these differences in men’s physical health narrowed as they grew older. Our findings on mental health are in line with the Chinese research [16] but contradict studies from the United States [18, 40]. This divergence may reflect contextual differences in family support and intergenerational intimacy in later life. In Western developed countries with well-established social security systems, older adults may depend less on their children for daily support and wellbeing. In China, by contrast, older parents often continue to rely heavily on their children for various forms of support, a pattern captured by the long-standing norm of *Yang Er Fang Lao*. For example, as parents grow older, their need for emotional companionship, financial assistance, and help in accessing healthcare services may increase. Better-educated children are generally more capable of providing such support because they tend to have greater economic resources, higher health literacy, and broader social networks. Empirical studies have shown that well-educated children can provide more financial and emotional support, promote healthier behaviors among parents, reduce parental stress, and enhance parents’ subjective social status and psychological wellbeing [15, 35]. By contrast, parents whose children have lower levels of education may receive more limited support and may be more exposed to stress related to their children’s unemployment or debt [16]. These disadvantages may become increasingly consequential in later life, thereby contributing to widening health disparities. In addition, cross-cultural differences in intergenerational intimacy may provide another explanation. Chinese family culture places strong emphasis on intergenerational responsibility and close parent–child ties, whereas many Western societies place greater emphasis on intimacy within the marital relationship [50, 51]. Such differences may shape the extent to which children’s educational resources are translated into parental health advantages. Consistent with this interpretation, recent national evidence indicates that 94.16% of older adults in China still prefer to age either in their own homes or in their children’s homes, and that about 80% of adult children provide financial support to their older parents [52].

For physical health, however, higher children’s education appeared to provide sustained benefits only for women’s IADL, while it did not slow age-related deterioration in men’s physical functioning. This gender difference may partly reflect women’s greater reliance on children’s educational resources due to their historically disadvantaged social position [10, 15]. Although women’s social status in China has improved in recent decades, the mothers in our sample belong mainly to cohorts whose lives were shaped by earlier inequalities in schooling, employment opportunities, and family responsibilities. These accumulated disadvantages may leave them more dependent on family-based resources in later life. At the same time, gendered patterns of intergenerational relationships may provide an additional explanation. Mothers often maintain closer emotional ties and more frequent contact with adult children than fathers [10], which may make children’s educational attainment more readily translated into support for mothers.

We also found that the association between children’s education and parental health tended to be weaker among more recent cohorts. One possible explanation is that older cohorts in China were more strongly embedded in family-centered systems of support, under which adult children played a central role in providing financial assistance, daily care, and health-related support to aging parents. The weaker association among more recent cohorts may instead reflect broader social transformation, including changing parent–child relationships, shifting family norms, and the improvement of formal support systems [51]. In addition, rapid educational expansion may have reduced the relative advantage associated with children’s schooling in younger cohorts, thereby weakening its marginal contribution to parental health. As a result, parental health in more recent cohorts may be shaped less directly by children’s educational resources than that of earlier generations.

Our findings reveal gender-specific protective effects of family members’ education on later-life health trajectories. For men, mental health trajectories benefited from spousal and children’s education, but physical health did not benefit from family members’ educational levels. This suggests that, despite high educational attainment providing men with significant material resources, their psychological wellbeing mainly depends on emotional support from other family members, especially their spouses. However, resources linked to family members’ education do not slow men’s physical health decline in old age, where biological aging mainly drives deterioration. For women, mental health trajectories benefited from the education of all family members, with their own and their children’s education being particularly influential, while physical health benefits came from their own and their children’s education. This may be due to Chinese women’s disadvantaged social position and their prominent roles as caregivers and kin-keepers, which increase their reliance on and potential to access health-related resources from their own and other family members’ education.

This study advances both empirical and theoretical insights into educational heterogeneity in later-life health trajectories. Empirically, we examine the educational attainment across three generations within a unified analytical framework, thereby providing a more comprehensive picture of multigenerational educational influences on health trajectories. We further compare these associations across physical and mental health outcomes and between men and women, offering a more nuanced understanding of heterogeneity in these relationships. Finally, by drawing on nearly a decade of longitudinal data from 2011 to 2020, we are able not only to capture more recent health patterns, but also, more importantly, to reduce the confounding influence of cohort effects in the analysis of health trajectories.

Theoretically, this study extends prior research by situating multigenerational education within a life-course and family stratification perspective, highlighting that educational resources from different family members may jointly and differentially shape later-life health. Furthermore, this study broadens the cumulative advantage/disadvantage hypothesis and the age-as-leveler hypothesis by examining their relevance to the associations between family members’ education and health trajectories. Our findings suggest that the applicability of these hypotheses depends on the type of health outcome considered and the gender examined. In addition, by testing these relationships in China, a rapidly changing developing-country context, this study calls into question assumptions derived primarily from Western settings. Prior studies in Western societies have shown that health disparities associated with children’s education tend to diminish with age [18, 31]. In our analysis of China, however, the opposite pattern emerged. This divergence may reflect several contextual differences. Notably, compared with many Western societies, China is characterized by stronger family interdependence and more enduring intergenerational obligations, which may allow children’s educational resources to exert a more lasting influence on parental health. Moreover, family support continues to play a particularly important role in later life in China, where welfare provision and formal care systems remain less developed in some respects. These contextual features may help explain why findings established in Western settings do not fully apply to China. More broadly, our results underscore the need for a context-sensitive understanding of how multigenerational educational resources shape health trajectories over the life course.

### Limitations

This study still has several limitations. First, it does not explore the mechanisms linking family members’ education to health trajectories. For instance, pathways such as coping strategies to adversities may mediate the impact of parental education on health, even after parents’ death. Second, the study does not consider the potential interactions between these educational influences. For example, it was unclear whether the effects of other family members’ education diminish with higher own education. However, this is beyond the scope of this study. And case numbers within analytic cells will be too small for a robust analysis if we further disaggregate data by gender, age, cohort, and the combination of family members’ education. Third, we do not distinguish the roles of maternal versus paternal education or sons’ versus daughters’ education in shaping health trajectories. And our main analyses measured parental education as the higher educational attainment of either parent and children’s education as the highest educational attainment among all children. This approach may not fully reflect the average or cumulative educational resources of all parents or all children. However, additional robustness analyses using average parental education and average children’s education yielded substantively similar conclusions. Future research could further compare the highest, average, and gender-specific measures of family members’ education to better distinguish different dimensions of intergenerational educational resources. Another limitation concerns the comparison between physical and mental health outcomes. Although IADL and CESD were standardized to place them on a common statistical scale, they capture different underlying constructs and have different measurement properties. Therefore, comparisons across these two domains should be interpreted cautiously. Finally, the CHARLS data, spanning 2011 to 2020, only offer a 9-year observation window per cohort, limiting generalizability to broader later-life trajectories and necessitating cautious interpretation when extrapolating trends.

### Conclusions

This study contributes to research on education and health by utilizing longitudinal data and a multi-generational approach to elucidate health trajectory patterns shaped by family members’ education, with variations across health indicators and genders in the Chinese context. These findings emphasize the importance of considering multiple generations, different health dimensions, and gender in health disparity research. Future research should explore mediating mechanisms, incorporate objective health measures, and perform comparative studies with other Asian countries. Policies promoting educational access for women and support for disadvantaged families are critical to reducing health inequalities in China’s aging population.

## Data Availability

Publicly available datasets were analysed in this study. This data can be found here: https://charls.pku.edu.cn/en/.

## References

[1] Mirowsky J Ross CE . Education, social status, and health. New York: Aldine De Gruyter (2003). p. 242.

[2] Ross CE Wu C . The links between education and health. Am Sociol Rev (1995) 60(5):719–45. 10.2307/2096319

[3] Leopold L Engelhartdt H . Education and physical health trajectories in old age. Evidence from the survey of health, ageing and retirement in Europe (SHARE). Int J Public Health (2013) 58(1):23–31. 10.1007/s00038-012-0399-0 22918517

[4] Hu A . Can education make us healthier? An urban-rural comparative analysis based on the Chinese general social survey of 2010. Soc Sci China (2015) 36(1):64–82. 10.1080/02529203.2015.1001321

[5] Halpern-Manners A Hernandez EM Wilbur TG . Crossover effects of education on health within married couples. J Health Soc Behav (2022) 63(2):301–18. 10.1177/00221465211063879 35001695

[6] Kim J Durden E . Socioeconomic status and age trajectories of health. Soc Sci Med (2007) 65(12):2489–502. 10.1016/j.socscimed.2007.07.022 17765375

[7] Lynch SM . Cohort and life-course patterns in the relationship between education and health: a hierarchical approach. Demography (2003) 40(2):309–31. 10.1353/dem.2003.0016 12846134

[8] Chen G Olsen JA Lamu AN . The influence of parents’ and partner’s education on own health behaviours. Soc Sci Med (2024) 343:116581. 10.1016/j.socscimed.2024.116581 38242029

[9] Gutierrez S Meza E Glymour MM Torres JM . My parent, myself, or my child: whose education matters Most for trajectories of cognitive aging in middle age? Am J Epidemiol (2023) 193:kwad108–706. 10.1093/aje/kwad108 37116072 PMC11484617

[10] Ma M Yahirun J Saenz J Sheehan C . Offspring educational attainment and older parents’ cognition in Mexico. Demography (2021) 58(1):75–109. 10.1215/00703370-8931725 33612872 PMC7894606

[11] Zeki AHA Haan MN Galea S Aiello AE . Life-course exposure to early socioeconomic environment, education in relation to late-life cognitive function among older Mexicans and Mexican Americans. J Aging Health (2011) 23(7):1027–49. 10.1177/0898264311421524 21948769 PMC3412879

[12] Luo L Wei L . For whom does education convey health benefits? A two-generation and life course approach. J Health Soc Behav (2024) 4:00221465241249120–617. 10.1177/00221465241249120 38832718 PMC11622520

[13] Brown DC Hummer RA Hayward MD . The importance of spousal education for the self-rated health of married adults in the United States. Popul Res Policy Rev (2014) 33(1):127–51. 10.1007/s11113-013-9305-6 24511172 PMC3912877

[14] Monden CWS Van Lenthe F De Graaf ND Kraaykamp G . Partner’s and own education: does who you live with matter for self-assessed health, smoking and excessive alcohol consumption? Soc Sci Med (2003) 57(10):1901–12. 10.1016/S0277-9536(03)00055-8 14499514

[15] Lee C . Adult children’s education and physiological dysregulation among older parents. J Gerontol Ser B (2018) 73(6):1143–54. 10.1093/geronb/gbx044 28444349 PMC6093314

[16] Li S Zhang S . The dynamic effect between offspring’s education attainment and the mental health of their parents: a longitudinal analysis with growth curve model. Popul & Development (2023) 29(1):123–36.

[17] Reynolds A Greenfield EA . Diminished returns of higher parental education on cognition for black adults in middle and later life. J Gerontol Ser B (2024) 79(3):gbad181. 10.1093/geronb/gbad181 38134238 PMC10878240

[18] Yahirun JJ Sheehan CM Mossakowski KN . Depression in later life: the role of adult children’s college education for older parents’ mental health in the United States. J Gerontol Ser B (2020) 75(2):389–402. 10.1093/geronb/gby135 30412237 PMC7530494

[19] Dannefer D . Cumulative advantage/disadvantage and the life course: cross-fertilizing age and social science theory. J Gerontol Ser B (2003) 58(6):S327–37. 10.1093/geronb/58.6.S327 14614120

[20] House JS Kessler RC Herzog AR . Age, socioeconomic status, and health. Milbank Q (1990) 68(3):383–411. 10.2307/3350111 2266924

[21] Yang F Jiang Y . Heterogeneous influences of social support on physical and mental health: evidence from China. Int J Environ Res Public Health (2020) 17(18):18. 10.3390/ijerph17186838 32962140 PMC7558190

[22] Leopold L . Cumulative advantage in an Egalitarian country? Socioeconomic health disparities over the life course in Sweden. J Health Soc Behav (2016) 57(2):257–73. 10.1177/0022146516645926 27284078

[23] Leopold L . Health measurement and health inequality over the life course: a comparison of self-rated health, SF-12, and grip strength. Demography (2019) 56(2):763–84. 10.1007/s13524-019-00761-x 30838536 PMC6449289

[24] Lee C Glei DA Goldman N Weinstein M . Children’s education and parents’ trajectories of depressive symptoms. J Health Soc Behav (2017) 58(1):86–101. 10.1177/0022146517690200 28661765 PMC5579841

[25] Qiu L Li J . Social differentiation of adult depression in China: a dynamic and intersectional perspective. Sociological Stud (2023) 38(5):180–202.

[26] Bian F Logan JR . A comparative analysis of intergenerational relations in China and United States. Sociological Res (2001) 16(2):85–95.

[27] Peng X Hu Z . The contemporary transition of the Chinese family and the reconstruction of family policy. Social Sci China (2015) 36(12):113–32.

[28] Yahirun JJ Vasireddy S Hayward MD . The education of multiple family members and the life-course pathways to cognitive impairment. J Gerontol Ser B (2020) 75(7):e113–28. 10.1093/geronb/gbaa039 32215643 PMC7424275

[29] Chen F Yang Y Liu G . Social change and socioeconomic disparities in health over the life course in China: a cohort analysis. Am Sociol Rev (2010) 75(1):126–50. 10.1177/0003122409359165 20379373 PMC2850448

[30] Zhao Y Hu Y Smith JP Strauss J Yang G . Cohort profile: the china health and retirement longitudinal study (CHARLS). Int J Epidemiol (2014) 43(1):61–8. 10.1093/ije/dys203 23243115 PMC3937970

[31] Friedman EM Mare RD . The schooling of offspring and the survival of parents. Demography (2014) 51(4):1271–93. 10.1007/s13524-014-0303-z 24917296

[32] Andresen EM Malmgren JA Carter WB Patrick DL . Screening for depression in well older adults: evaluation of a short form of the CES-D. Am J Prev Med (1994) 10(2):77–84. 10.1016/S0749-3797(18)30622-6 8037935

[33] Hu Y Xu Y Wang G . The regularity in the elder’s health status transition and its implication on disability prevention. Chin J Popul Sci (2024) 38(2):19–34.

[34] Tang T Jiang J Tang X . Prevalence of depressive symptoms among older adults in mainland China: a systematic review and meta-analysis. J Affect Disord (2021) 293:379–90. 10.1016/j.jad.2021.06.050 34246000

[35] Pei Y Cong Z Wu B . Education, adult children’s education, and depressive symptoms among older adults in rural China. Soc Sci Med (2020) 253:112966. 10.1016/j.socscimed.2020.112966 32247217

[36] Zhang S Li S . Intergenerational educational mobility and the difference of mental health of middle-aged and elder parents. J Xi'an Jiaotong Univ (Social Sciences) (2022) 42(6):121–32.

[37] Ge T Van Leeuwen FJ Jiang Q Leopold L . Mental health in China: social change in life course trajectories. Popul Dev Rev (2024) 51:759–96. 10.1111/padr.12684

[38] Raftery AE . Bayesian model selection in social research. Sociol Methodol (1995) 25:111–63. 10.2307/271063

[39] Von Hippel PT . 4. Regression with missing ys: an improved strategy for analyzing multiply imputed data. Sociol Methodol (2007) 37(1):83–117. 10.1111/j.1467-9531.2007.00180.x

[40] Yahirun J Sheehan C Mossakowski K . Black–white differences in the link between offspring college attainment and parents’ depressive symptom trajectories. Res Aging (2022) 44(2):123–35. 10.1177/0164027521997999 33678079 PMC8423861

[41] O’Brien R . Age-period-cohort models: approaches and analyses with aggregate data. Boca Raton: CRC Press (2015).

[42] Ross CE Mirowsky J . The interaction of personal and parental education on health. Soc Sci Med (2011) 72(4):591–9. 10.1016/j.socscimed.2010.11.028 21227556 PMC3049298

[43] Saenz JL Beam CR Zelinski EM . The association between spousal education and cognitive ability among older Mexican adults. J Gerontol Ser B (2020) 75(7):e129–40. 10.1093/geronb/gbaa002 31974544 PMC7424282

[44] Zheng L Zeng X . Cohort and age patterns in the relationship between education and health: an examination by gender and hukou. J East China Univ Sci Technology (Social Sci Edition) (2018) 33(2):54–65.

[45] Reuter M . A longitudinal analysis of health inequalities from adolescence to young adulthood and their underlying causes. Adv Life Course Res (2024) 59, 1–19. 10.1016/j.alcr.2024.100593 38340523

[46] Kalmijn M . Discrepancies in parents’ perceptions of adult children’s well-being: evidence from mother–father–child triads. J Fam Stud (2024) 30(5):838–60. 10.1080/13229400.2024.2335493 39319027 PMC11418899

[47] Xu S Zhang H Wang J . Caregiver burden and depression among Chinese family caregivers: the role of self-compassion. Mindfulness (2020) 11(7):1647–54. 10.1007/s12671-020-01378-7

[48] Angelini V Howdon DDH Mierau JO . Childhood socioeconomic status and late-adulthood mental health: results from the survey on health, ageing and retirement in Europe. J Gerontol B Psychol Sci Soc Sci (2019) 74(1):95–104. 10.1093/geronb/gby028 29566242 PMC6941210

[49] Assari S Cobb S Bazargan M . Parental education differently boosts health and happiness of American men and women. J Community Med Health Educ (2020) 10, 678–690. Available online at: https://pubmed.ncbi.nlm.nih.gov/34548955/. 34548955

[50] Yan Y . The private life approach to the rise of neo-familism in China. Sociol Rev (2025) 73(4):753–70. 10.1177/00380261251347745

[51] Zeng X Li Y . Transition and continuation: a typological study of intergenerational relationships in Chinese families. Chin J Sociol (2024) 10(1):76–99. 10.1177/2057150X241229032

[52] Du P Sun J Zhang W Wang X . The demands of old-age care and the family and social resources for the Chinese elderly: a study based on 2014 China longitudinal aging social survey. Popul Res (2016) 40(6):49–61.

