# Supplementary material for: Whose education matters for later-life health trajectories? A three-generation comparison in China
Source: Int J Public Health. 2026 May 20;71:1609298. doi: 10.3389/ijph.2026.1609298 (PMC13229840; doi:10.3389/ijph.2026.1609298)
Supplement: Supplementary file 1 [file Supplementaryfile1.docx]

**Supplemental materials**

**Contents**

**Table A1** Sample restrictions

**Table A2** Distribution of education levels across cohorts

**Table A3** Characteristics by missing status in family members’ education

**Table A4** Characteristics at the 2011 baseline by dropout status

**Table A5** Association between earlier health status and dropout at next wave among men

**Table A6** Association between earlier health status and dropout at next wave among women

**Table A7** Results of the hierarchical linear model for CESD trajectories

**Table A8** Results of the hierarchical linear model for IADL trajectories

**Table A9** Estimated 9-year change in own education differences in CESD and IADL, by gender and cohort

**Table A10** Estimated 9-year change in parental education differences in CESD and IADL, by gender and cohort

**Table A11** Estimated 9-year change in spousal education differences in CESD and IADL, by gender and cohort

**Table A12** Estimated 9-year change in children’s education differences in CESD and IADL, by gender and cohort

**Table A13** Definition and measurement of covariates

**Fig. A1** Observed CESD patterns

**Fig. A2** Observed IADL patterns

**Fig. A3** Predicted age trajectories of CESD-by-education: Control for health

**Fig. A4** Predicted age trajectories of IADL-by-education: Control for health

**Fig. A5** Observed patterns of CESD and IADL by period and cohort

**Fig. A6** Predicted age trajectories of CESD-by-education: Control for period

**Fig. A7** Predicted age trajectories of IADL-by-education: Control for period

**Fig. A8** Predicted age trajectories of CESD and IADL-by-education: Using parental average education level

**Fig. A9** Predicted age trajectories of CESD and IADL-by-education: Using the average education level of all children

**Sample Restriction**

**Table A1** Sample restrictions

| Restrictions | Observations | Individuals |
| --- | --- | --- |
| Original sample | 74,118 | 17,705 |
| Excluding |  |  |
| Age < 45 or age > 85 | 2,892 | 551 |
| Never married | 611 | 159 |
| No children aged ≥ 25 | 6,980 | 1,605 |
| Missing in IADL, gender and own education | 799 | 86 |
| Final sample | 62,836 | 15,304 |

*Notes:* IADL = Instrumental Activities of Daily Living (IADL).

**Education Distribution**

**Table A2** Distribution of education levels across cohorts

| Original education | 10-year cohort | | | | | | | | |
| --- | --- | --- | --- | --- | --- | --- | --- | --- | --- |
|  | 1900 ^a^ | 1910 | 1920 | 1930 | 1940 | 1950 | 1960 | 1970 | 1980 |
| *Panel A: Cut-off values for low, medium, and high education* | | | | | | | | | |
| No formal education | Low | Low | Low | Low | Low | Low | Low | Low | Low |
| Capable of reading or writing | Med | Med | Med | Med | Low | Low | Low | Low | Low |
| Elementary school | High | High | High | Med | Med | Low | Low | Low | Low |
| Middle school | High | High | High | High | High | Med | Med | Low | Low |
| High school | High | High | High | High | High | High | High | Med | Med |
| Two/Three-year college | High | High | High | High | High | High | High | High | Med |
| Bachelor’s degree and above | High | High | High | High | High | High | High | High | High |
| *Panel B: Percentage of people with low, medium, and high education (%)* | | | | | | | | | |
| Lower education | 72.03 | 68.51 | 59.07 | 47.40 | 49.28 | 64.46 | 40.08 | 60.23 | 51.02 |
| Medium education | 18.37 | 21.08 | 23.83 | 39.10 | 28.22 | 21.72 | 34.12 | 21.10 | 32.40 |
| Higher education | 9.60 | 10.41 | 17.10 | 13.49 | 22.49 | 13.82 | 25.80 | 18.67 | 16.57 |

*Notes*: ^a^ Birth cohorts of 1900 and earlier. Low = Low education. Med = Medium education. High = High education.

**Observed Patterns**

**Fig. A1** Observed CESD patterns

*Notes:* CESD = Depressive symptoms. In each panel, red lines show the observed patterns of people with low education, while the black lines show the observed patterns of people with high education.

**Fig. A2** Observed IADL patterns

*Notes:* IADL = Instrumental Activities of Daily Living. In each panel, red lines show the observed patterns of people with low education, while the black lines show the observed patterns of people with high education.

**Missing Analyses**

**Table A3** Characteristics by missing status in family members’ education and CESD

| Variables | Not missing | | | | | Missing ^a^ | | | | | Differences *(p*-value) ^b^ | |
| --- | --- | --- | --- | --- | --- | --- | --- | --- | --- | --- | --- | --- |
|  | Mean / % | | SD | | | Mean / % | | SD | | |  |  |
| *Panel A: Missing in other family members’ education* | | | | | | | | | | | | |
| Gender |  | |  | | |  | |  | | | 0.001 | |
| Men | 46.79% | |  | | | 48.72% | |  | | |  | |
| Women | 53.21% | |  | | | 51.28% | |  | | |  | |
| Own education |  | |  | | |  | |  | | | 0.000 | |
| Low | 58.70% | |  | | | 48.42% | |  | | |  | |
| Medium | 26.12% | |  | | | 29.65% | |  | | |  | |
| High | 15.19% | |  | | | 21.93% | |  | | |  | |
| Age | 64.20 | | 8.49 | | | 59.19 | | 9.75 | | | 0.000 | |
| Birth year | 1950.85 | | 8.16 | | | 1955.80 | | 9.48 | | | 0.000 | |
| CESD | 8.72 | | 6.46 | | | 7.86 | | 6.18 | | | 0.000 | |
| IADL | 6.17 | | 2.79 | | | 5.84 | | 2.43 | | | 0.000 | |
| *Panel B: Missing in CESD* | | | | | | | | | | | | |
| Gender | |  | |  | | |  | |  | | | 0.009 |
| Men | | 47.26% | |  | | | 45.56% | |  | | |  |
| Women | | 52.74% | |  | | | 54.44% | |  | | |  |
| Own education | |  | |  | | |  | |  | | | 0.000 |
| Low | | 56.17% | |  | | | 65.66% | |  | | |  |
| Medium | | 27.21% | |  | | | 21.81% | |  | | |  |
| High | | 16.62% | |  | | | 12.53% | |  | | |  |
| Parental education | |  | |  | | |  | |  | | | 0.000 |
| Low | | 61.74% | |  | | | 68.88% | |  | | |  |
| Medium | | 25.58% | |  | | | 21.48% | |  | | |  |
| High | | 12.68% | |  | | | 9.64% | |  | | |  |
| Spousal education | |  | |  | | |  | |  | | | 0.000 |
| Low | | 55.85% | |  | | | 59.13% | |  | | |  |
| Medium | | 27.40% | |  | | | 26.90% | |  | | |  |
| High | | 16.75% | |  | | | 13.97% | |  | | |  |
| Children’s education | |  | |  | | |  | |  | | | 0.040 |
| Low | | 50.70% | |  | | | 51.04% | |  | | |  |
| Medium | | 29.29% | |  | | | 27.93% | |  | | |  |
| High | | 20.01% | |  | | | 21.03% | |  | | |  |
| Age | | 63.06 | | | 8.57 | | 66.83 | | | 10.46 | | 0.000 |
| Birth year | | 1951.94 | | 8.31 | | | 1948.52 | | 9.80 | | | 0.000 |
| IADL | | 5.88 | | | 2.23 | | 8.19 | | | 4.94 | | 0.000 |

*Notes:* ^a^ Missing refers to those who had missing values in family members’ education (i.e., parental, spousal and children’s education) or CESD. ^b^ *Chi-square* test for categorical variables and *t* test for continuous variables were conducted to examine the difference by missing status in sample characteristics. Non-standardized values of CESD and IADL were used. CESD = Depressive symptoms. IADL = Instrumental Activities of Daily Living. SD = Standard Deviation.

**Selective Attrition Analyses**

**Table A4** Characteristics at the 2011 baseline by dropout status

| Variables | Not dropout | | Dropout ^a^ | | Differences *(p*-value) ^b^ |
| --- | --- | --- | --- | --- | --- |
|  | Mean / % | SD | Mean / % | SD |  |
| Gender |  |  |  |  | 0.000 |
| Men | 45.95 |  | 53.48 |  |  |
| Women | 54.05 |  | 46.52 |  |  |
| Own education |  |  |  |  | 0.000 |
| Low | 58.06 |  | 50.29 |  |  |
| Medium | 26.12 |  | 28.64 |  |  |
| High | 15.81 |  | 21.08 |  |  |
| Parental education |  |  |  |  | 0.000 |
| Low | 62.18 |  | 64.26 |  |  |
| Medium | 25.57 |  | 22.39 |  |  |
| High | 12.25 |  | 13.35 |  |  |
| Spousal education |  |  |  |  | 0.000 |
| Low | 56.20 |  | 54.22 |  |  |
| Medium | 27.54 |  | 25.97 |  |  |
| High | 16.25 |  | 19.80 |  |  |
| Children’s education |  |  |  |  | 0.000 |
| Low | 51.33 |  | 42.20 |  |  |
| Medium | 28.82 |  | 30.57 |  |  |
| High | 19.85 |  | 27.23 |  |  |
| Age | 58.79 | 8.26 | 64.38 | 10.55 | 0.000 |
| Birth year | 1952.21 | 8.26 | 1946.62 | 10.55 | 0.000 |
| CESD | 8.41 | 6.33 | 8.92 | 6.57 | 0.000 |
| IADL | 5.68 | 1.89 | 6.85 | 3.76 | 0.000 |

*Notes:* ^a^ Dropout refers to those who participated in the 2011 baseline did not participant in the last wave. ^b^ *Chi-square* test for categorical variables and *t* test for continuous variables were conducted to examine the gender difference in sample characteristics. Non-standardized values of CESD and IADL were used. CESD = Depressive symptoms. IADL = Instrumental Activities of Daily Living. SD = Standard Deviation.

**Table A5** Association between earlier health status and dropout at next wave among men

| Variables | Drop at wave 2 | | Drop at wave 3 | | Drop at wave 4 | | Drop at wave 5 | |
| --- | --- | --- | --- | --- | --- | --- | --- | --- |
|  | β | SE | β | SE | β | SE | β | SE |
| CESD | 0.018 | (0.015) | 0.014 | (0.017) | 0.010 | (0.014) | -0.017 | (0.015) |
| IADL | 0.051^**^ | (0.016) | 0.104^***^ | (0.017) | 0.107^***^ | (0.015) | 0.150^***^ | (0.016) |
| Age | -0.004 | (0.003) | 0.003 | (0.004) | 0.002 | (0.003) | 0.006 | (0.003) |
| Own education (ref. Low) | | | | | | | | |
| Medium | -0.038 | (0.079) | 0.090 | (0.089) | -0.087 | (0.077) | -0.042 | (0.084) |
| High | -0.259^**^ | (0.099) | 0.140 | (0.109) | 0.121 | (0.094) | -0.055 | (0.106) |
| Medium # Age | -0.001 | (0.004) | -0.002 | (0.004) | 0.005 | (0.004) | 0.005 | (0.004) |
| High # Age | 0.011^*^ | (0.005) | -0.007 | (0.005) | -0.005 | (0.004) | 0.004 | (0.005) |
| Parental education (ref. Low) | | | | | | | | |
| Medium | 0.034 | (0.080) | 0.033 | (0.087) | 0.089 | (0.078) | -0.240^**^ | (0.086) |
| High | 0.212^*^ | (0.101) | 0.261^*^ | (0.117) | 0.077 | (0.103) | 0.041 | (0.115) |
| Medium # Age | -0.004 | (0.004) | -0.003 | (0.004) | -0.009^*^ | (0.004) | 0.008^*^ | (0.004) |
| High # Age | -0.007 | (0.005) | -0.016^**^ | (0.006) | -0.004 | (0.005) | -0.004 | (0.006) |
| Spousal education (ref. Low) | | | | | | | | |
| Medium | 0.042 | (0.085) | 0.279^**^ | (0.091) | -0.059 | (0.083) | 0.162 | (0.090) |
| High | 0.160 | (0.112) | 0.737^***^ | (0.117) | 0.130 | (0.115) | 0.215 | (0.124) |
| Medium # Age | 0.000 | (0.004) | -0.007 | (0.004) | 0.002 | (0.004) | -0.004 | (0.004) |
| High # Age | 0.003 | (0.005) | -0.022^***^ | (0.006) | 0.001 | (0.005) | -0.000 | (0.006) |
| Children’s education (ref. Low) | | | | | | | | |
| Medium | 0.211^**^ | (0.080) | -0.197^*^ | (0.091) | -0.090 | (0.077) | -0.354^***^ | (0.087) |
| High | 0.217^*^ | (0.096) | 0.058 | (0.101) | -0.175 | (0.096) | -0.350^**^ | (0.107) |
| Medium # Age | -0.008 | (0.004) | 0.011^**^ | (0.004) | 0.008^*^ | (0.004) | 0.016^***^ | (0.004) |
| High # Age | -0.004 | (0.005) | 0.012^**^ | (0.005) | 0.011^**^ | (0.004) | 0.016^***^ | (0.005) |
| Constant | -1.494^***^ | (0.065) | -1.819^***^ | (0.075) | -1.414^***^ | (0.062) | -1.537^***^ | (0.067) |
| Pseudo *R*^2^ | 0.009 |  | 0.027 |  | 0.011 |  | 0.028 |  |

*Notes:* Drop refers to those who participated in the 2011 baseline did not participant in the last wave. CESD = Depressive symptoms. IADL = Instrumental Activities of Daily Living. SE = Standard errors. ^*^ *p* < 0.05, ^**^ *p* < 0.01, ^***^ *p* < 0.001

**Table A6** Association between earlier health status and dropout at next wave among women

| Variables | Drop at wave 2 | | Drop at wave 3 | | Drop at wave 4 | | Drop at wave 5 | |
| --- | --- | --- | --- | --- | --- | --- | --- | --- |
|  | β | SE | β | SE | β | SE | β | SE |
| CESD | 0.012 | (0.013) | -0.010 | (0.014) | -0.038^**^ | (0.013) | -0.014 | (0.013) |
| IADL | 0.038^*^ | (0.015) | 0.052^**^ | (0.017) | 0.124^***^ | (0.014) | 0.126^***^ | (0.015) |
| Age | 0.000 | (0.003) | 0.010^**^ | (0.003) | -0.001 | (0.003) | 0.003 | (0.003) |
| Own education (ref. Low) | | | | | | | | |
| Medium | 0.072 | (0.076) | 0.169^*^ | (0.082) | -0.019 | (0.079) | 0.317^***^ | (0.080) |
| High | 0.122 | (0.103) | 0.464^***^ | (0.105) | 0.152 | (0.113) | 0.454^***^ | (0.108) |
| Medium # Age | -0.002 | (0.004) | -0.002 | (0.004) | -0.004 | (0.004) | -0.009^*^ | (0.004) |
| High # Age | 0.002 | (0.005) | -0.003 | (0.005) | -0.008 | (0.006) | -0.007 | (0.005) |
| Parental education (ref. Low) | | | | | | | | |
| Medium | -0.120 | (0.074) | -0.070 | (0.079) | -0.073 | (0.074) | -0.166^*^ | (0.082) |
| High | 0.098 | (0.090) | 0.136 | (0.101) | 0.180 | (0.100) | 0.330^***^ | (0.094) |
| Medium # Age | 0.001 | (0.004) | 0.004 | (0.004) | 0.006 | (0.004) | -0.001 | (0.004) |
| High # Age | -0.001 | (0.005) | -0.005 | (0.005) | -0.012^*^ | (0.005) | -0.011^*^ | (0.005) |
| Spousal education (ref. Low) | | | | | | | | |
| Medium | 0.038 | (0.070) | 0.291^***^ | (0.078) | -0.032 | (0.072) | -0.072 | (0.076) |
| High | -0.066 | (0.087) | 0.246^*^ | (0.096) | 0.050 | (0.087) | 0.066 | (0.093) |
| Medium # Age | -0.004 | (0.004) | -0.015^***^ | (0.004) | 0.002 | (0.004) | 0.004 | (0.004) |
| High # Age | 0.002 | (0.004) | -0.014^**^ | (0.005) | 0.003 | (0.004) | -0.002 | (0.005) |
| Children’s education (ref. Low) | | | | | | | | |
| Medium | 0.234^**^ | (0.073) | 0.056 | (0.081) | 0.015 | (0.074) | -0.327^***^ | (0.079) |
| High | 0.549^***^ | (0.082) | 0.442^***^ | (0.089) | 0.133 | (0.087) | -0.230^*^ | (0.094) |
| Medium # Age | -0.005 | (0.004) | -0.002 | (0.004) | 0.007 | (0.004) | 0.019^***^ | (0.004) |
| High # Age | -0.011^**^ | (0.004) | -0.006 | (0.004) | 0.007 | (0.004) | 0.017^***^ | (0.004) |
| Constant | -1.670^***^ | (0.058) | -1.980^***^ | (0.066) | -1.614^***^ | (0.058) | -1.678^***^ | (0.060) |
| Pseudo *R*^2^ | 0.016 |  | 0.030 |  | 0.020 |  | 0.029 |  |

*Notes:* Drop refers to those who participated in the 2011 baseline did not participant in the last wave. CESD = Depressive symptoms. IADL = Instrumental Activities of Daily Living. SE = Standard errors. ^*^ *p* < 0.05, ^**^ *p* < 0.01, ^***^ *p* < 0.001

**Main Analyses**

**Table A7** Results of the hierarchical linear model for CESD trajectories

| Variables | Own education | | | | Parental education | | | | Spousal education | | | | Children’s education | | | |
| --- | --- | --- | --- | --- | --- | --- | --- | --- | --- | --- | --- | --- | --- | --- | --- | --- |
|  | M1: Men | | M2: Women | | M3: Men | | M4: Women | | M5: Men | | M6: Women | | M7: Men | | M8: Women | |
|  | β | SE | β | SE | β | SE | β | SE | β | SE | β | SE | β | SE | β | SE |
| Age | -0.035^*^ | (0.015) | -0.013 | (0.015) | -0.035^*^ | (0.015) | -0.017 | (0.015) | -0.038^**^ | (0.015) | -0.016 | (0.015) | -0.028 | (0.015) | -0.013 | (0.015) |
| Age^2^ | 0.001^***^ | (0.000) | 0.000^*^ | (0.000) | 0.001^***^ | (0.000) | 0.001^*^ | (0.000) | 0.001^***^ | (0.000) | 0.001^*^ | (0.000) | 0.001^**^ | (0.000) | 0.001^*^ | (0.000) |
| Cohort | -0.010 | (0.006) | 0.004 | (0.006) | -0.012 | (0.006) | -0.000 | (0.006) | -0.014^*^ | (0.006) | 0.000 | (0.007) | -0.010 | (0.006) | 0.003 | (0.006) |
| Age # Cohort | 0.001^***^ | (0.000) | 0.001^**^ | (0.000) | 0.001^***^ | (0.000) | 0.001^***^ | (0.000) | 0.001^***^ | (0.000) | 0.001^***^ | (0.000) | 0.001^**^ | (0.000) | 0.001^**^ | (0.000) |
| Own education (ref. Low) | | | | | | | | | | | | | | | | |
| Medium | 0.084 | (0.170) | -0.044 | (0.192) | -0.095^***^ | (0.021) | -0.169^***^ | (0.025) | -0.095^***^ | (0.021) | -0.169^***^ | (0.025) | -0.093^***^ | (0.021) | -0.167^***^ | (0.026) |
| High | -0.281 | (0.180) | 0.339 | (0.225) | -0.221^***^ | (0.025) | -0.367^***^ | (0.032) | -0.221^***^ | (0.025) | -0.366^***^ | (0.032) | -0.217^***^ | (0.025) | -0.365^***^ | (0.032) |
| Medium # Age | -0.003 | (0.004) | -0.002 | (0.004) |  |  |  |  |  |  |  |  |  |  |  |  |
| High # Age | 0.001 | (0.004) | -0.014^**^ | (0.005) |  |  |  |  |  |  |  |  |  |  |  |  |
| Medium # Cohort | -0.005 | (0.004) | -0.003 | (0.005) |  |  |  |  |  |  |  |  |  |  |  |  |
| High # Cohort | 0.002 | (0.005) | -0.017^**^ | (0.006) |  |  |  |  |  |  |  |  |  |  |  |  |
| Parental education (ref. Low) | | | | | | | | | | | | | | | | |
| Medium | -0.029 | (0.022) | -0.049^*^ | (0.024) | -0.021 | (0.169) | 0.004 | (0.186) | -0.029 | (0.022) | -0.049^*^ | (0.024) | -0.028 | (0.022) | -0.048^*^ | (0.024) |
| High | -0.053 | (0.028) | -0.069^*^ | (0.033) | 0.075 | (0.221) | 0.101 | (0.247) | -0.054 | (0.028) | -0.070^*^ | (0.033) | -0.052 | (0.028) | -0.070^*^ | (0.033) |
| Medium # Age |  |  |  |  | -0.003 | (0.004) | -0.004 | (0.004) |  |  |  |  |  |  |  |  |
| High # Age |  |  |  |  | -0.005 | (0.005) | -0.006 | (0.005) |  |  |  |  |  |  |  |  |
| Medium # Cohort |  |  |  |  | 0.001 | (0.004) | 0.000 | (0.005) |  |  |  |  |  |  |  |  |
| High # Cohort |  |  |  |  | -0.002 | (0.006) | -0.003 | (0.006) |  |  |  |  |  |  |  |  |
| Spousal education (ref. Low) | | | | | | | | | | | | | | | | |
| Medium | -0.048 | (0.027) | -0.037 | (0.025) | -0.049 | (0.027) | -0.037 | (0.025) | -0.359 | (0.192) | 0.036 | (0.182) | -0.046 | (0.027) | -0.037 | (0.025) |
| High | -0.090^**^ | (0.031) | -0.098^**^ | (0.031) | -0.091^**^ | (0.031) | -0.097^**^ | (0.031) | -0.065 | (0.219) | -0.011 | (0.215) | -0.090^**^ | (0.031) | -0.096^**^ | (0.031) |
| Medium # Age |  |  |  |  |  |  |  |  | 0.006 | (0.004) | -0.004 | (0.004) |  |  |  |  |
| High # Age |  |  |  |  |  |  |  |  | -0.003 | (0.005) | -0.001 | (0.004) |  |  |  |  |
| Medium # Cohort |  |  |  |  |  |  |  |  | 0.008 | (0.005) | -0.000 | (0.005) |  |  |  |  |
| High # Cohort |  |  |  |  |  |  |  |  | 0.001 | (0.006) | -0.002 | (0.006) |  |  |  |  |
| Children’s education (ref. Low) | | | | | | | | | | | | | | | | |
| Medium | -0.090^***^ | (0.024) | -0.127^***^ | (0.024) | -0.090^***^ | (0.024) | -0.129^***^ | (0.023) | -0.090^***^ | (0.024) | -0.128^***^ | (0.023) | -0.045 | (0.169) | 0.308 | (0.189) |
| High | -0.174^***^ | (0.025) | -0.202^***^ | (0.027) | -0.174^***^ | (0.025) | -0.199^***^ | (0.027) | -0.173^***^ | (0.025) | -0.200^***^ | (0.027) | -0.348 | (0.188) | -0.076 | (0.206) |
| Medium # Age |  |  |  |  |  |  |  |  |  |  |  |  | -0.004 | (0.004) | -0.010^*^ | (0.004) |
| High # Age |  |  |  |  |  |  |  |  |  |  |  |  | -0.003 | (0.004) | -0.007 | (0.005) |
| Medium # Cohort |  |  |  |  |  |  |  |  |  |  |  |  | 0.001 | (0.004) | -0.010^*^ | (0.005) |
| High # Cohort |  |  |  |  |  |  |  |  |  |  |  |  | 0.008 | (0.005) | -0.001 | (0.005) |
| No spouse | 0.292^***^ | (0.040) | 0.173^***^ | (0.031) | 0.291^***^ | (0.040) | 0.172^***^ | (0.031) | 0.291^***^ | (0.040) | 0.172^***^ | (0.031) | 0.288^***^ | (0.040) | 0.171^***^ | (0.031) |
| No parents alive | 0.041 | (0.022) | 0.030 | (0.024) | 0.040 | (0.022) | 0.030 | (0.024) | 0.041 | (0.022) | 0.030 | (0.024) | 0.042 | (0.022) | 0.032 | (0.024) |
| Constant | 0.175 | (0.248) | -0.060 | (0.246) | 0.214 | (0.243) | 0.062 | (0.251) | 0.305 | (0.237) | 0.046 | (0.253) | 0.149 | (0.236) | -0.079 | (0.242) |
| Observations | 24031 |  | 26466 |  | 24031 |  | 26466 |  | 24031 |  | 26466 |  | 24031 |  | 26466 |  |

*Notes*: CESD = Depressive symptoms. SE = Standard errors. ^*^ *p* < 0.05, ^**^ *p* < 0.01, ^***^ *p* < 0.001

**Table A8** Results of the hierarchical linear model for IADL trajectories

| Variables | Own education | | | | Parental education | | | | Spousal education | | | | Children’s education | | | |
| --- | --- | --- | --- | --- | --- | --- | --- | --- | --- | --- | --- | --- | --- | --- | --- | --- |
|  | M9: Men | | M10: Women | | M11: Men | | M12: Women | | M13: Men | | M14: Women | | M15: Men | | M16: Women | |
|  | β | | SE | | β | | SE | | β | | SE | | β | | SE | |
| Age | -0.011^***^ | (0.003) | -0.002 | (0.003) | -0.010^***^ | (0.003) | 0.000 | (0.003) | -0.012^***^ | (0.003) | -0.001 | (0.003) | -0.013^***^ | (0.003) | -0.001 | (0.003) |
| Age^2^ | 0.001^***^ | (0.000) | 0.001^***^ | (0.000) | 0.001^***^ | (0.000) | 0.001^***^ | (0.000) | 0.001^***^ | (0.000) | 0.001^***^ | (0.000) | 0.001^***^ | (0.000) | 0.001^***^ | (0.000) |
| Cohort | -0.008^**^ | (0.003) | -0.003 | (0.002) | -0.004^*^ | (0.002) | -0.001 | (0.002) | -0.005^**^ | (0.002) | -0.005 | (0.003) | -0.007^***^ | (0.002) | -0.003 | (0.003) |
| Own education (ref. Low) | | | | | | | | | | | | | | | | |
| Medium | -0.224 | (0.147) | -0.369^*^ | (0.163) | -0.056^***^ | (0.014) | -0.076^***^ | (0.018) | -0.056^***^ | (0.014) | -0.076^***^ | (0.018) | -0.054^***^ | (0.014) | -0.073^***^ | (0.018) |
| High | -0.324^*^ | (0.159) | -0.212 | (0.180) | -0.074^***^ | (0.016) | -0.132^***^ | (0.019) | -0.073^***^ | (0.016) | -0.133^***^ | (0.019) | -0.072^***^ | (0.016) | -0.132^***^ | (0.019) |
| Medium # Age | 0.001 | (0.003) | 0.004 | (0.004) |  |  |  |  |  |  |  |  |  |  |  |  |
| High # Age | 0.001 | (0.004) | -0.004 | (0.004) |  |  |  |  |  |  |  |  |  |  |  |  |
| Medium # Cohort | 0.005 | (0.004) | 0.008 | (0.004) |  |  |  |  |  |  |  |  |  |  |  |  |
| High # Cohort | 0.008^*^ | (0.004) | 0.004 | (0.004) |  |  |  |  |  |  |  |  |  |  |  |  |
| Parental education (ref. Low) | | | | | | | | | | | | | | | | |
| Medium | -0.028 | (0.015) | -0.025 | (0.015) | -0.088 | (0.142) | 0.009 | (0.148) | -0.028 | (0.015) | -0.025 | (0.015) | -0.028 | (0.015) | -0.024 | (0.015) |
| High | -0.017 | (0.022) | -0.017 | (0.022) | -0.077 | (0.190) | 0.144 | (0.200) | -0.017 | (0.022) | -0.016 | (0.022) | -0.017 | (0.022) | -0.016 | (0.022) |
| Medium # Age |  |  |  |  | -0.001 | (0.003) | -0.002 | (0.003) |  |  |  |  |  |  |  |  |
| High # Age |  |  |  |  | -0.000 | (0.004) | -0.005 | (0.004) |  |  |  |  |  |  |  |  |
| Medium # Cohort |  |  |  |  | 0.002 | (0.004) | -0.000 | (0.004) |  |  |  |  |  |  |  |  |
| High # Cohort |  |  |  |  | 0.002 | (0.005) | -0.003 | (0.005) |  |  |  |  |  |  |  |  |
| Spousal education (ref. Low) | | | | | | | | | | | | | | | | |
| Medium | -0.017 | (0.016) | -0.025 | (0.017) | -0.018 | (0.016) | -0.026 | (0.017) | -0.260 | (0.159) | -0.205 | (0.160) | -0.016 | (0.016) | -0.024 | (0.017) |
| High | -0.026 | (0.016) | -0.054^**^ | (0.019) | -0.025 | (0.016) | -0.055^**^ | (0.019) | -0.127 | (0.195) | -0.259 | (0.176) | -0.024 | (0.016) | -0.054^**^ | (0.019) |
| Medium # Age |  |  |  |  |  |  |  |  | 0.004 | (0.004) | -0.001 | (0.004) |  |  |  |  |
| High # Age |  |  |  |  |  |  |  |  | 0.002 | (0.005) | 0.001 | (0.004) |  |  |  |  |
| Medium # Cohort |  |  |  |  |  |  |  |  | 0.006 | (0.004) | 0.006 | (0.004) |  |  |  |  |
| High # Cohort |  |  |  |  |  |  |  |  | 0.003 | (0.005) | 0.006 | (0.004) |  |  |  |  |
| Children’s education (ref. Low) | | | | | | | | | | | | | | | | |
| Medium | -0.027 | (0.017) | -0.078^***^ | (0.016) | -0.027 | (0.017) | -0.078^***^ | (0.015) | -0.026 | (0.017) | -0.077^***^ | (0.015) | -0.309^*^ | (0.146) | -0.051 | (0.153) |
| High | -0.036 | (0.019) | -0.086^***^ | (0.021) | -0.038 | (0.019) | -0.088^***^ | (0.021) | -0.038 | (0.019) | -0.086^***^ | (0.021) | -0.268 | (0.167) | -0.179 | (0.178) |
| Medium # Age |  |  |  |  |  |  |  |  |  |  |  |  | 0.006 | (0.004) | -0.003 | (0.003) |
| High # Age |  |  |  |  |  |  |  |  |  |  |  |  | 0.003 | (0.004) | -0.003 | (0.004) |
| Medium # Cohort |  |  |  |  |  |  |  |  |  |  |  |  | 0.007^*^ | (0.004) | 0.000 | (0.004) |
| High # Cohort |  |  |  |  |  |  |  |  |  |  |  |  | 0.006 | (0.004) | 0.004 | (0.004) |
| No spouse | -0.006 | (0.024) | -0.034 | (0.021) | -0.003 | (0.024) | -0.032 | (0.021) | -0.002 | (0.024) | -0.035 | (0.021) | -0.003 | (0.024) | -0.033 | (0.021) |
| No parents alive | 0.011 | (0.013) | 0.009 | (0.015) | 0.011 | (0.013) | 0.007 | (0.015) | 0.011 | (0.013) | 0.008 | (0.015) | 0.011 | (0.013) | 0.009 | (0.015) |
| Constant | 0.045 | (0.109) | -0.032 | (0.087) | -0.064 | (0.084) | -0.134 | (0.092) | -0.023 | (0.077) | 0.021 | (0.115) | 0.053 | (0.088) | -0.063 | (0.105) |
| Observations | 24031 |  | 26466 |  | 24031 |  | 26466 |  | 24031 |  | 26466 |  | 24031 |  | 26466 |  |

*Notes*: IADL = Instrumental Activities of Daily Living. SE = Standard errors. ^*^ *p* < 0.05, ^**^ *p* < 0.01, ^***^ *p* < 0.001

**Table A9** Estimated 9-year change in own education differences in CESD and IADL, by gender and cohort

| Age at first wave  (Birth cohort) | CESD | | | |  | IADL | | | |
| --- | --- | --- | --- | --- | --- | --- | --- | --- | --- |
|  | Men | | Women | |  | Men | | Women | |
|  | Initial  difference | 9 years  later | Initial  difference | 9 years  later |  | Initial  difference | 9 years  later | Initial  difference | 9 years  later |
| 46 | 0.209^***^ | 0.203^***^ | 0.355^***^ | 0.485^***^ |  | 0.014 | 0.006 | 0.060^*^ | 0.098^**^ |
| (1965) | [0.124,0.294] | [0.110,0.296] | [0.258,0.453] | [0.377,0.593] |  | [-0.034,0.062] | [-0.057,0.069] | [0.010,0.110] | [0.031,0.165] |
| 56 | 0.220^***^ | 0.214^***^ | 0.325^***^ | 0.455^***^ |  | 0.084^***^ | 0.076^*^ | 0.143^***^ | 0.181^***^ |
| (1955) | [0.165,0.276] | [0.145,0.284] | [0.256,0.394] | [0.367,0.542] |  | [0.048,0.121] | [0.016,0.135] | [0.099,0.187] | [0.113,0.248] |
| 66 | 0.232^***^ | 0.226^***^ | 0.295^***^ | 0.424^***^ |  | 0.154^***^ | 0.145^***^ | 0.225^***^ | 0.263^***^ |
| (1945) | [0.170,0.294] | [0.149,0.303] | [0.206,0.383] | [0.317,0.531] |  | [0.092,0.215] | [0.064,0.227] | [0.152,0.298] | [0.171,0.356] |
| 76 | 0.243^***^ | 0.237^***^ | 0.264^***^ | 0.394^***^ |  | 0.223^***^ | 0.215^***^ | 0.308^***^ | 0.346^***^ |
| (1935) | [0.145,0.341] | [0.127,0.347] | [0.128,0.401] | [0.242,0.546] |  | [0.125,0.321] | [0.100,0.329] | [0.195,0.420] | [0.218,0.474] |
| Averages for all cohorts | 0.226 | 0.220 | 0.310 | 0.440 |  | 0.119 | 0.111 | 0.184 | 0.222 |
| Average change |  | -0.006 |  | +0.130 |  |  | -0.008 |  | +0.038 |

*Notes:* Estimates are average marginal differences in standard deviations between low- and high-education groups based on M1 and M2 in Table A7, and M9 and M10 in Table A8. 95% confidence intervals are shown in brackets. Initial differences are predicted mean differences in CESD or IADL at the age of first observation in the survey. 9-years later differences are predicted mean differences in CESD or IADL at the age of last observation in the survey. Averages of initial and 9-year later differences were calculated for all cohorts. Average change was calculated as the average difference between predicted mean differences at initial observation and predicted mean differences 9 years later for all cohorts. Differences in gray shadow are not statistically significant (*p* > 0.05).

**Table A10** Estimated 9-year change in parental education differences in CESD and IADL, by gender and cohort

| Age at first wave  (Birth cohort) | CESD | | | |  | IADL | | | |
| --- | --- | --- | --- | --- | --- | --- | --- | --- | --- |
|  | Men | | Women | |  | Men | | Women | |
|  | Initial  difference | 9 years  later | Initial  difference | 9 years  later |  | Initial  difference | 9 years  later | Initial  difference | 9 years  later |
| 46 | 0.004 | 0.047 | 0.013 | 0.068 |  | -0.006 | -0.002 | -0.018 | 0.032 |
| (1965) | [-0.095,0.103] | [-0.061,0.154] | [-0.095,0.120] | [-0.048,0.184] |  | [-0.058,0.047] | [-0.079,0.075] | [-0.084,0.048] | [-0.049,0.112] |
| 56 | 0.032 | 0.075 | 0.047 | 0.102^*^ |  | 0.020 | 0.024 | 0.006 | 0.056 |
| (1955) | [-0.029,0.094] | [-0.008,0.158] | [-0.025,0.119] | [0.012,0.191] |  | [-0.030,0.070] | [-0.060,0.108] | [-0.043,0.055] | [-0.019,0.130] |
| 66 | 0.060 | 0.103 | 0.081 | 0.136^*^ |  | 0.046 | 0.050 | 0.030 | 0.080 |
| (1945) | [-0.018,0.139] | [-0.000,0.206] | [-0.016,0.177] | [0.022,0.250] |  | [-0.054,0.145] | [-0.076,0.176] | [-0.049,0.109] | [-0.022,0.182] |
| 76 | 0.089 | 0.131 | 0.115 | 0.170^*^ |  | 0.072 | 0.076 | 0.054 | 0.104 |
| (1935) | [-0.042,0.219] | [-0.020,0.283] | [-0.040,0.269] | [0.001,0.339] |  | [-0.086,0.230] | [-0.104,0.255] | [-0.072,0.181] | [-0.041,0.249] |
| Averages for all cohorts | 0.046 | 0.089 | 0.064 | 0.119 |  | 0.033 | 0.037 | 0.018 | 0.068 |
| Average change |  | +0.043 |  | +0.055 |  |  | +0.004 |  | +0.050 |

*Notes:* Estimates are average marginal differences in standard deviations between low- and high-education groups based on M3 and M4 in Table A7, and M11 and M12 in Table A8. 95% confidence intervals are shown in brackets. Initial differences are predicted mean differences in CESD or IADL at the age of first observation in the survey. 9-years later differences are predicted mean differences in CESD or IADL at the age of last observation in the survey. Averages of initial and 9-year later differences were calculated for all cohorts. Average change was calculated as the average difference between predicted mean differences at initial observation and predicted mean differences 9 years later for all cohorts. Differences in gray shadow are not statistically significant (*p* > 0.05).

**Table A11** Estimated 9-year change in spousal education differences in CESD and IADL, by gender and cohort

| Age at first wave  (Birth cohort) | CESD | | | |  | IADL | | | |
| --- | --- | --- | --- | --- | --- | --- | --- | --- | --- |
|  | Men | | Women | |  | Men | | Women | |
|  | Initial  difference | 9 years  later | Initial  difference | 9 years  later |  | Initial  difference | 9 years  later | Initial  difference | 9 years  later |
| 46 | 0.029 | 0.059 | 0.108 | 0.119 |  | 0.024 | 0.005 | 0.009 | 0.000 |
| (1965) | [-0.073,0.131] | [-0.051,0.169] | [-0.007,0.223] | [-0.009,0.247] |  | [-0.030,0.078] | [-0.064,0.075] | [-0.049,0.067] | [-0.071,0.072] |
| 56 | 0.073^*^ | 0.103^*^ | 0.096^**^ | 0.106^*^ |  | 0.029 | 0.010 | 0.063^**^ | 0.054 |
| (1955) | [0.008,0.137] | [0.012,0.194] | [0.027,0.164] | [0.020,0.193] |  | [-0.010,0.068] | [-0.069,0.089] | [0.020,0.106] | [-0.013,0.121] |
| 66 | 0.116^**^ | 0.147^*^ | 0.083^*^ | 0.094 |  | 0.034 | 0.015 | 0.117^**^ | 0.108^*^ |
| (1945) | [0.035,0.197] | [0.032,0.262] | [0.003,0.163] | [-0.001,0.188] |  | [-0.058,0.125] | [-0.112,0.141] | [0.033,0.201] | [0.005,0.211] |
| 76 | 0.160^*^ | 0.190^*^ | 0.070 | 0.081 |  | 0.038 | 0.019 | 0.171^*^ | 0.162^*^ |
| (1935) | [0.028,0.293] | [0.026,0.354] | [-0.065,0.206] | [-0.062,0.224] |  | [-0.116,0.192] | [-0.166,0.204] | [0.032,0.310] | [0.008,0.316] |
| Averages for all cohorts | 0.095 | 0.125 | 0.089 | 0.100 |  | 0.031 | 0.012 | 0.090 | 0.081 |
| Average change |  | +0.030 |  | +0.011 |  |  | -0.019 |  | -0.009 |

*Notes:* Estimates are average marginal differences in standard deviations between low- and high-education groups based on M5 and M6 in Table A7, and M13 and M14 in Table A8. 95% confidence intervals are shown in brackets. Initial differences are predicted mean differences in CESD or IADL at the age of first observation in the survey. 9-years later differences are predicted mean differences in CESD or IADL at the age of last observation in the survey. Averages of initial and 9-year later differences were calculated for all cohorts. Average change was calculated as the average difference between predicted mean differences at initial observation and predicted mean differences 9 years later for all cohorts. Differences in gray shadow are not statistically significant (*p* > 0.05).

**Table A12** Estimated 9-year change in children’s education differences in CESD and IADL, by gender and cohort

| Age at first wave  (Birth cohort) | CESD | | | |  | IADL | | | |
| --- | --- | --- | --- | --- | --- | --- | --- | --- | --- |
|  | Men | | Women | |  | Men | | Women | |
|  | Initial  difference | 9 years  later | Initial  difference | 9 years  later |  | Initial  difference | 9 years  later | Initial  difference | 9 years  later |
| 46 | 0.034 | 0.058 | 0.107^*^ | 0.167^**^ |  | 0.012 | -0.015 | 0.007 | 0.038 |
| (1965) | [-0.066,0.135] | [-0.038,0.153] | [0.008,0.205] | [0.068,0.266] |  | [-0.041,0.065] | [-0.085,0.056] | [-0.059,0.073] | [-0.040,0.116] |
| 56 | 0.141^***^ | 0.164^***^ | 0.167^***^ | 0.227^***^ |  | 0.047^*^ | 0.020 | 0.086^***^ | 0.117^***^ |
| (1955) | [0.079,0.203] | [0.096,0.233] | [0.109,0.226] | [0.151,0.304] |  | [0.006,0.088] | [-0.049,0.089] | [0.042,0.131] | [0.049,0.186] |
| 66 | 0.248^***^ | 0.271^***^ | 0.228^***^ | 0.288^***^ |  | 0.082^*^ | 0.055 | 0.166^***^ | 0.197^***^ |
| (1945) | [0.188,0.308] | [0.193,0.349] | [0.145,0.311] | [0.180,0.396] |  | [0.018,0.145] | [-0.035,0.144] | [0.097,0.235] | [0.105,0.289] |
| 76 | 0.355^***^ | 0.378^***^ | 0.288^***^ | 0.348^***^ |  | 0.116^*^ | 0.089 | 0.245^***^ | 0.276^***^ |
| (1935) | [0.259,0.451] | [0.263,0.493] | [0.144,0.433] | [0.181,0.516] |  | [0.016,0.217] | [-0.033,0.212] | [0.133,0.357] | [0.145,0.408] |
| Averages for all cohorts | 0.195 | 0.218 | 0.198 | 0.258 |  | 0.064 | 0.037 | 0.126 | 0.157 |
| Average change |  | +0.023 |  | +0.060 |  |  | -0.027 |  | +0.031 |

*Notes:* Estimates are average marginal differences in standard deviations between low- and high-education groups based on M7 and M8 in Table A7, and M15 and M16 in Table A8. 95% confidence intervals are shown in brackets. Initial differences are predicted mean differences in CESD or IADL at the age of first observation in the survey. 9-years later differences are predicted mean differences in CESD or IADL at the age of last observation in the survey. Averages of initial and 9-year later differences were calculated for all cohorts. Average change was calculated as the average difference between predicted mean differences at initial observation and predicted mean differences 9 years later for all cohorts. Differences in gray shadow are not statistically significant (*p* > 0.05).

**Controlling for Covariates**

**Table A13** Definition and measurement of covariates

| Variables | Definition | Measurement |
| --- | --- | --- |
| Hukou | The household registration status | 0 = rural; 1 = urban |
| Smoking | Smoking or not | 0 = no; 1 = yes |
| Drinking | Drink any alcoholic beverages or not | 0 = no; 1 = yes |
| Self-rated health before 15 years old | Self-comment of health status during childhood, up to and including age 15 | 0 = poor, fair;  1 = good, very good, excellent |
| Self-rated health | Self-comment of health status | 0 = very poor, poor, fair  1 = good, very good |
| Chronic diseases | Number of chronic diseases | 0 = no; 1 = at least one |
| IADL limitations | Number of activities that need help or unable to complete, including doing household chores, preparing hot meals, shopping for groceries, managing assets, and taking medications | 0 = no; 1 = at least one |
| Depression | Total score of the 10-item short form of the Center for Epidemiologic Studies Depression Scale | 0 = scored below 10  1 = scored 10 and above |

*Notes*: IADL = Instrumental Activities of Daily Living. All covariates were measured at the 2011 baseline.

**Fig. A3** Predicted age trajectories of CESD-by-education: Control for health

*Notes:* Due to space limitations, only the results controlling for health status are presented. CESD = Depressive symptoms. In each panel, black lines show the trajectories of people with low education, while gray lines show the trajectories of people with high education.

**Fig. A4** Predicted age trajectories of IADL-by-education: Control for health

*Notes:* Due to space limitations, only the results controlling for health status are presented. IADL = Instrumental Activities of Daily Living. In each panel, black lines show the trajectories of people with low education, while gray lines show the trajectories of people with high education.

**Period Effects**

**Fig. A5** Observed patterns of CESD and IADL by period and cohort

*Notes:* CESD = Depressive symptoms. IADL = Instrumental Activities of Daily Living.

**Fig. A6** Predicted age trajectories of CESD-by-education: Control for period

*Notes:* CESD = Depressive symptoms. In each panel, black lines show the trajectories of people with low education, while gray lines show the trajectories of people with high education.

**Fig. A7** Predicted age trajectories of IADL-by-education: Control for period

*Notes:* IADL = Instrumental Activities of Daily Living. In each panel, black lines show the trajectories of people with low education, while gray lines show the trajectories of people with high education.

**Using Parental Average Education Level**

**Fig. A8** Predicted age trajectories of CESD and IADL-by-education: Using parental average education level

*Notes:* CESD = Depressive symptoms. IADL = Instrumental Activities of Daily Living. In each panel, black lines show the trajectories of people with low education, while gray lines show the trajectories of people with high education.

**Using Children’s Average Education Level**

**Fig. A9** Predicted age trajectories of CESD and IADL-by-education: Using the average education level of all children

*Notes:* CESD = Depressive symptoms. IADL = Instrumental Activities of Daily Living. In each panel, black lines show the trajectories of people with low education, while gray lines show the trajectories of people with high education.
